# Insecurity and psychological well-being among faculty in academia: exploring the constraints and conduits of positive psychological functioning

**DOI:** 10.1080/17482631.2025.2474361

**Published:** 2025-04-03

**Authors:** Anna S. Tanimoto, Johanna Segerbäck, Anne Richter, Petra Lindfors

**Affiliations:** a Department of Psychology, Stockholm University, Stockholm, Sweden; b Department of Learning, Informatics, Management and Ethics, Karolinska Institutet, Stockholm, Sweden

**Keywords:** Quantitative job insecurity, qualitative job insecurity, eudaimonia, psychological well-being, faculty, higher education institutions

## Abstract

**Purpose:**

Job insecurity characterizes academic work, with potential risks for the health, well-being, and personal lives of faculty. Notwithstanding, faculty with job insecurity experiences may still find academia conducive to pursuing personal fulfilment. As faculty experiences of psychological well-being may be coloured by insecurity, this study sought to qualitatively investigate the ways in which experiences of insecurity and psychological well-being co-occur.

**Methods:**

This study followed a questionnaire study of a representative sample of faculty in Swedish academia and their job insecurity perceptions, inviting the most insecure to participate. The participant group included 19 faculty from nine public Swedish higher education institutions. Transcripts of the semi-structured interviews were analysed using reflexive thematic analysis, guided by the six theoretical dimensions of psychological well-being.

**Results:**

Two themes were developed: 1) Staying afloat?, and 2) I’m not yet where I’m supposed to be. These themes elucidate faculty experiences of managing their current work (and personal) situations, and reveal how faculty orient themselves in relation to their futures, pasts and presents.

**Conclusions:**

The findings demonstrate how experiences of insecurity co-exist with psychological well-being in constraining and enhancing faculty well-being. This reveals how psychological well-being involves a dynamic process of negotiation, especially during transitional periods.

Academia is increasingly characterized by issues related to employment and working conditions. Concerns pertaining to job insecurity are ubiquitous and experiences of insecurity may be negatively associated with faculty health and well-being, even encroach upon their personal lives (Nicholls et al., 2022). Despite such negative implications of job insecurity for faculty, academia may provide opportunities for faculty to pursue personal fulfilment with potential for psychological well-being (PWB). Indeed, opportunities to contribute to society and experience a sense of autonomy may underlie faculty motivation to pursue academia and academic work (e.g., Pautz & Vogel, 2020). Such possible benefits, yet also various constraints related to working in academia make it important to explore experiences of insecurity and PWB among faculty. As research expressly focusing on PWB in terms of individual resources from a qualitative approach is limited, qualitatively mapping how faculty experiences of insecurity relate to PWB has the potential to yield important insights into faculty experiences which facilitate and hinder individual fulfilment.

## Psychological well-being

While PWB is often conceptualized as the *absence* of mental health complaints (Bautista et al., 2023), it can be framed as a construct embodying positive psychological functioning (Keyes et al., 2002). Research focusing on overall PWB and positive psychological functioning is generally rooted in hedonic and eudaimonic perspectives. Hedonic well-being is typically equated with happiness, connoting the presence of pleasure and positive affect, and the absence of negative affect, whereas eudaimonia refers to the pursuit of living in accordance with one’s potential, often defined as the affective state of an individual striving to pursue this (Ryan & Deci, 2001; Ryff, 1989). As a global construct emphasizing eudaimonic well-being, while acknowledging the key role of positive affect, PWB can be considered to include six theoretically defined dimensions, each representing a facet of individual resources central to positive psychological functioning (Ryff, 1989). Table I provides a brief overview of these dimensions.

**Table I.** The six psychological well-being dimensions and their definitions (e.g., Ryff, 1989).

PWB Dimension	Description
Self-acceptance	An individual’s acknowledgement and acceptance of both positive and negative aspects of their past and present life.
Positive relations with others	A capacity for empathy as well as having and maintaining trusting relationships.
Autonomy	The extent to which an individual acts independently and is able to resist social pressures.
Environmental mastery	A sense of capability and competence to manage and make use of one’s surrounding environment(s).
Purpose in life	A sense of directedness and belief that there is meaning in life.
Personal growth	An interest in continued self-development and openness to change, new experiences, and expansion.

Various contexts and situations, including work settings and work tasks, provide individuals with opportunities to pursue and develop the individual resources covered by the six PWB dimensions (e.g., Soren & Ryff, 2023). Consequently, there is a need to understand what work stressors, involving insecurities related to one’s job, may imply for individual experiences of positive psychological functioning, including PWB. This is especially relevant for highly educated individuals who may identify strongly with their work (Miron et al., 2022).

## Defining insecurity

Literature on employment and working conditions in academia show the ubiquity of job insecurity, by focusing on temporary employment (e.g., Castellacci & Viñas-Bardolet, 2021) commonly referred to as *objective insecurity*. Other studies investigate *subjective job insecurity* (e.g., Urbanaviciute et al., 2021), focusing on individuals’ perceptions of their work situation regardless of their employment contract. Subjective job insecurity covers two dimensions, namely *quantitative* and *qualitative* insecurity. The former refers to the perceived threat of job loss, whereas the latter regards the perceived threat of deteriorating working conditions (Hellgren et al., 1999). Faculty experiences of job insecurity may extend beyond such operationalizations and include a multitude of insecurities. Specifically, their experiences may stem from the interplay of various distinct aspects of their work, including the amount of time allocated to teaching versus research, the success of grant applications, and conceptions about the external labour market (Segerbäck, 2023). To broadly account for the job insecurity-related perceptions of faculty, it seems reasonable to refer to these as *insecurity*.

## Aim

Job insecurity is considered a work stressor with potentially detrimental effects, and recent studies indicate that high job insecurity is associated with poorer self-reported well-being among faculty (Tanimoto et al., 2025; Urbanaviciute et al., 2021), and white-collar workers (Låstad et al., 2021). However, current job insecurity research is based predominantly on quantitative studies. Qualitative approaches may bring new insights to the current understanding of job insecurity, specifically how individuals think about, make sense of, and experience job insecurity in various contexts (Bazzoli & Probst, 2022; Klug et al., 2024). For instance, faculty experiences and expressions of insecurity and well-being, particularly PWB, remain largely understudied from a qualitative perspective. Thus, what is known and what is lacking motivates the current research. Consequently, the present qualitative study used fine-grained accounts to investigate faculty experiences of insecurity and PWB, contributing to a context-specific empirical understanding, specifically, in academia. This study set out to answer the following research question:

How is psychological well-being experienced and expressed by faculty with experiences of insecurity in academia?

## Methodology

### Participant selection

This interview study served as a qualitative follow-up to a questionnaire study conducted in 2021 in collaboration with Statistics Sweden. For that questionnaire study, a representative sample of faculty, with a doctoral degree, employed at one of 30 public higher education institutions (HEIs) in Sweden were invited (*n* = 7446) and 39% responded. After excluding those combining work in academia with retirement, parental leave, or other similar activities, the study consisted of 2729 faculty. Respondents were asked to indicate whether they could be contacted later for an interview study and if so, were prompted to provide contact information. Approximately 50% of respondents gave their permission.

A latent profile analysis, where respondents were statistically categorized into groups based on their concurrent perceptions of quantitative and qualitative job insecurity, resulted in five distinctive *profiles* of job insecurity^1^ (Tanimoto et al., 2025). One profile, the *Insecure*, was found the most vulnerable regarding job insecurity perceptions and self-ratings on health-related indicators (including a measure of hedonic and eudaimonic well-being). In an effort to better understand experiences of high insecurity in academia, and how such experiences occur in relation to well-being, interviewees were recruited exclusively from this profile, which contained 73 potential volunteers. Those who provided contact information were contacted either via email or telephone, and provided with information about the study. To assess eligibility, the potential respondents answered questions regarding their employment status and their perceived job insecurity. The job insecurity questions were identical to those in the 2021 questionnaire study (Fischmann et al., 2022; Låstad et al., 2015). Individuals were *not* eligible if a) they no longer worked in a Swedish HEI, b) were on full-time parental leave, c) did not indicate moderate to high job insecurity,^2^ or d) no longer wished to participate. If the eligibility criteria were met, an invitation to the interview was extended.

### Participant group^3^

The current study includes 19 individuals (eight women, eleven men). Their ages ranged from 32 to 62, and the extent of their employment ranged from 25% of full-time to 100^4^ percent. They were employed at nine public HEIs across Sweden, with various employment situations including permanent, fixed-term, and *permanent; dependent on funding* (Swedish Association of University Teachers and Researchers & National Junior Faculty, 2021). Ethical approval for this study was granted by the Swedish Ethical Review Authority (diary number: 2020–07195).

### Data collection

The dataset consisted of 19 semi-structured interviews that were conducted digitally over Zoom between October 2022 and February 2023. Each interview took approximately one hour. The majority of the interviews (more than 70%) were conducted in Swedish by a native speaker (JS): the rest were conducted in English, also by a native speaker (AST). The language used in an interview was determined based on interviewee preferences.

Each interview followed the same procedure. Starting with a short overview of the study, including details on the handling of personal details and information about informed consent, interviewees were then given the possibility to ask questions. Next, interviewees were asked to describe and reflect upon their current employment situations. Specifically, they were prompted to describe their work situation in terms of their position, what their employment contract entailed in terms of teaching and research, to reflect on their work environment, and overall to share how they viewed their work situation. Most provided short descriptions of their career trajectories, starting from when they received their doctoral degrees, ending with their current employment. Interviewees were allowed to speak freely until a new prompt or follow-up question was needed to encourage further reflections or sharing of experiences. When possible, they were asked to describe instances which exemplified general experiences or opinions expressed.

### Data preparation

Each interview was audio-recorded, transcribed verbatim and then quality-checked. Next, the interviews were de-identified one at a time. This process involved reading through each interview and masking information. For instance, detailed information about an interviewee’s health status and other personal data was reworked (summarized with the details removed) or removed entirely when there was a potential risk of revealing their identity. However, whenever possible, information was reformulated to maintain a clear and coherent text. In most cases, participants’ employment positions and academic disciplines were retained. To maintain interviewee integrity, some transcripts were edited for grammatical errors which otherwise would have revealed a non-native speaker of the interview language. Finally, the fully de-identified transcripts were quality-controlled by AST and PL to ensure that the material was usable while also protecting interviewees’ integrity.

### Positionality and choice of analytical approach

A critical realism approach was adopted using reflexive thematic analysis (RTA; Braun & Clarke, 2006, 2022). In RTA, patterns of shared meaning across a dataset are shaped and developed by the researcher, who takes an active role in meaning-making (Braun & Clarke, 2006, 2022; Finlay, 2021). Not only is RTA flexible in allowing for inductive and deductive approaches to analysis, but it acknowledges and embraces the subjectivity of the researcher whose knowledge and expertise are harnessed as an asset to the analytical process, given their prior theoretical and empirical knowledge relevant to the subject area under investigation. Given this, it is important to acknowledge the researcher’s *position* as this may contribute to an increased understanding of their situated interpretation and analysis of the material (Levitt et al., 2017). Here, AST conducted the analysis and is currently a doctoral candidate with more than three years of experience of researching faculty in Swedish academia. Not only does AST have empirical and anecdotal knowledge about the experiences of faculty in Sweden, but also they are in the unique position of writing a dissertation about the conditions of their future, should they strive to pursue an academic career. Moreover, AR and PL have their own experiences of Swedish academia, given their more senior positions and academic tenure. Thus, the accumulated knowledge and experience would be impossible to ignore, so RTA was selected as a suitable analytical approach which would put such knowledge to use.

RTA follows six analytical phases, namely 1) data familiarization, 2) coding, 3) generating initial themes, 4) developing and reviewing themes, 5) refining, defining, naming themes, and 6) writing up (Braun & Clarke, 2022). Due to the reflexive and recursive characteristics of RTA, the researcher is encouraged to continuously question and interrogate their findings and the analysis as it progresses, which ultimately involves returning to prior phases in a recursive process.

### Data analysis

Data familiarization involved listening to, transcribing and de-identifying the interview material. Coding was conducted by AST, and Ryff’s six PWB dimensions (1989) helped guide the deductive coding process, with descriptions of high and low scorers on the various dimensions used as inspiration to identify expressions of PWB. Some codes were labelled without a particular dimension in mind, reflecting an inductive approach to allow for content that seemed potentially relevant to the research question, but lacking a straightforward, theoretically driven categorization. Throughout the coding process, examples of coded segments and codes were collated and discussed between AST and PL to ensure that the interpretations made sense, even if other interpretations were also possible. A recommended minimum of two rounds of coding and recoding were conducted (Braun & Clarke, 2022). Phases three and four involved developing themes and subthemes, reviewing themes in relation to data segments and consolidation (see Table II for an example of how coded data segments contributed to theme development). Themes were named and defined in phases five and six, which involved articulating the characteristics of each theme and subtheme, using relevant data extracts, and ultimately writing up the report.

**Table II.** Two examples of a coded data segment and how it contributed to theme development.

Data segment	Code	Subtheme	Theme
[…] I try when I’m at home to put my work phone away so I don’t hear it ringing. I try to be very strict about not looking at emails or answering emails when I am off work or trying to unwind. (5019)	Boundary-setting: do not engage	Acts of resistance	Staying afloat?
[…] now it’s obvious that there’s someone else who needs my time and my attention and my love and everything and before that it didn’t exist and then yes, that in itself can create an imbalance. (5010)	Evolving priorities	Downward and upward adjustment	I’m not yet where I’m supposed to be

## Results

To answer the research question, *how psychological well-being is experienced and expressed by faculty with experiences of insecurity in academia*, two themes were developed. These elucidate how faculty create meaning and understanding of their own experiences while occupying a position^5^ of insecurity. The first theme, *Staying afloat?*, encompasses the factors and forces that contribute to faculty experiences of *sinking* and *buoyancy*. The second theme, *I’m not yet where I’m supposed to be*, covers how faculty make sense of their positions of liminality: how they orient themselves in relation to their futures, pasts and presents. Both themes differentiate experiences of faculty in space and experiences of faculty in time: a time of transition, between insecurity and security.

### Staying afloat?

*Staying afloat?* exemplifies the efforts and struggles expressed by faculty to manage their current situations: within the professional domain (i.e., in their academic roles and their related duties), within the personal domain (including family and health), and to reconcile responsibilities between domains. These efforts and struggles operate within the formal and informal constraints and opportunities of academia, as well as within the constraints and opportunities of the personal circumstances (i.e., family responsibilities, routines, etc.). Staying afloat? evokes the metaphor of a swimmer trying to endure rough waters, sometimes treading water, sometimes sinking beneath the surface, and sometimes kicking until resurfacing again. It includes four subthemes: 1.1) *“It’s about trying not to get totally burnt out”*, 1.2) *others can determine my career path*, 1.3) *acts of resistance, and*, 1.4) *my work as a source of meaning*.

1.1. The subtheme *“It’s about trying not to get totally burnt out”* refers to faculty efforts to keep up with their work responsibilities. Managing these responsibilities is not an isolated endeavour as these efforts have direct implications for other aspects of their lives. This theme accounts for how faculty spend their time, and their approaches to managing. One such approach is by working more. Faculty workloads require more than the typical 40-hour weeks, yet, working more is still not enough. Further compounding this dilemma is the awareness that these efforts to manage can go too far; faculty must actively ensure that they avoid getting totally burnt out, which requires monitoring one’s work-related stress, noticing changes related to one’s health:

Now I’ve just started to exercise a little and walk in the morning instead so now I’m starting to feel a little better which is good. But no, so it’s been like a vicious spiral that you don’t have time to exercise ((-)) and then you get more and more tired and don’t have time. But yeah, now I’m, now I’m on my way trying to find find some time for it. (5021)

Prior experiences of health complaints affect the ways that faculty approach managing their current situations.

I still feel like I have to work too much and it’s affected my physical health ((-)) and it’s something that I haven’t had time to take care of because I have this big project that I’m finishing right now [-] (5004).

Other solutions for managing not only the workload, but avoiding full-blown burnout involve a practice of reallocating time and sacrifice.

Managing involves a practice of time reallocation. As time is finite, working overtime means that the time to do so must ultimately come from elsewhere. Faculty practice time reallocation, where non-work time^6^ (i.e., evenings, weekends, and vacations) is needed and used to fulfil professional responsibilities. In these reallocation practices, there is also sacrifice. Here, a faculty member articulates their practice of time reallocation, involving feelings of obligation; not only to deliver *research*, but to sacrifice their vacation time in order to carve out the necessary time. Their experience elicits a belief of having “stolen” time from another department from which they receive research time, but which time consistently gets used up by work for a different department.

[-] so actually what suffers, it’s my research above all which meant that I was forced to take a shorter vacation for example and do research [-] (5001).

To make things right, they must borrow time from themselves (i.e., vacation time), but whether this time is ever repaid remains unclear.

While reallocation is about time and ultimately sacrificing time from other temporal domains, sacrifices are more nuanced. These involve the quality and/or quantity of relationships with others, including family members or friends. In fact, faculty share the common experience that having social lives is a question of time, leaving little time for social relationships outside the nuclear family.

Other sacrifices entail aspects pertaining to one’s health or health behaviours, such as exercise and sleep routines:

what’s most problematic is that I think there’s less time that I can spend to kind of you know, exercise, things like that. (5013)

While these sacrifices may appear to be temporary to manage one’s current situation, there is always the risk that in conjunction with time reallocation, faculty put themselves at a greater risk for burnout and potentially jeopardize resources necessary for managing.

1.2. The second subtheme, *others can determine my career path*, refers to the ways in which faculty express having internalized the formal and informal structures of academia in efforts for continuity—ultimately to be able to stay in academia. Formal structures involve constraints related to employment legislation, funding-related regulations, and departmental rules for recruitment and promotion, for instance. Given that teaching experience and student evaluations are considered in faculty evaluations for promotion, a formal constraint regards the necessity of good student evaluations.

Making a good impression on students is clearly important for promotion, but also there is a clear pattern across the material which speaks to the informal structures or norms in academia which seem to *require* faculty to make a good impression on others so as not to jeopardize their future academic prospects. These informal requirements include expressions of needing to come across as reliable, cooperative, and as someone who avoids causing trouble:

[-] I felt that, if I’d felt secure in my employment, I would have taken it further, even if it’s only a small sum of money, but I think it’s about all of us, everyone, and here I felt that, no, I don’t want to argue because then they will be able to make sure that they don’t schedule me at all next semester because I have no right to these hours either. (5022)

This example underscores a process of constant self-regulation, where faculty must weigh the potential impact of their actions (or inactions), and regulate themselves in ways that do not jeopardize their academic futures.

Faculty share experiences where formal and informal constraints are at play. One interviewee expresses these constraints on their autonomy—in this case, to be able to *attempt* to do research—as both informal and formal, where the formal constraint is embedded in the informal:

the last few years I’ve worked my ass off trying to create a foundation at several different institutions at the same time so that they’ll see me as important enough to apply for research funding from there. [-] (5018)

Good collegial relationships are considered a necessity to be able to apply for research grants, and without strong professional connections, research opportunities are curtailed.

Building and maintaining professional relationships are portrayed both as informal constraints and as opportunities. An important informal structure for faculty, highlighted across the material, refers to their experiences of having a colleague(s), usually more senior faculty, who look out for them and their professional interests. These *mentors* offer expert advice, they advocate on behalf of faculty, and they help to provide opportunities for learning and development.

1.3. An important facet of managing or trying to manage regards faculty *acts of resistance*, the third subtheme, referring to the efforts faculty make to assert or gain a sense of individual control over their situations, connoting a sense of agency, or *buoyancy*. These acts can be seen as the various tools faculty make use of in their efforts to make their situations more manageable, through carving out opportunities to exert control or discretion. These tools include boundary-setting, devising individual strategies evoking a sense of control, and seeking out the social support of others, for instance.

Notably, faculty speak of boundaries in spatial and temporal terms, but also as a kind of deviation from the norms of academia. Spatial boundaries are drawn by closing doors and changing one’s environment. Temporal boundaries stipulate when individuals choose to engage with work, and to what extent (i.e., how many hours). Embedded in such concrete examples, in articulating these boundaries, lies a more latent resistance. Faculty mark their individuality, their agency, in relation to academic norms and discourse about academic work.

Specifically, distancing as a form of resistance contributes to a sense of buoyancy among faculty in that they create and cultivate their *own* beliefs, standards, and expectations for themselves regarding what it means to be an academic or to work in academia, rather than conceding to the existing norms or discourse of academia:

[-] I’m not focused on external factors, like I’m not in academia to be winner of some prize (5027).

Faculty also acknowledge the consequences which arise from these acts of resistance. In one case, by *resisting*, (publishing in open access journals as opposed to those with paywalls), their academic portfolio was not competitive enough to receive funding as the publications were not in high impact journals. Thus, some acts of resistance may work against faculty interests of continuity, even if they provide respite (in terms of perceived control) in a space characterized by feelings of a lack of control.

Another latent *act of resistance* lies embedded in examples of faculty policing themselves, specifically their negativity or their *complaining* throughout the interviews. Faculty draw attention to the extent to which they voice the negative aspects of their experiences and current situations in academia, without focusing “enough” on the positive:

now I’ve only talked about what’s bad but, I belong to those who, I sound like a whiner, I’m incredibly privileged, I belong to an elite who gets large grants to do research with what I love and I get to choose to some extent my collaborations and everything so that I have it, I have it really good on the other hand I have it really bad, it sounds really strange but there are always two extremes here, so I sound very spoiled. [-] (5009)

This resistance is multi-layered in that faculty have internalized the academic discourse of constructive criticism (as opposed to complaining), and “recognize it” in some way as complaining. Still, they break with this discourse, or academic tradition, voicing their complaints, speaking out, and doing so in a space of empirical inquiry. This lends legitimacy to their “complaining”. Interestingly, while these instances do reflect some degree of faculty policing themselves, calling attention to “complaining” is another way in which faculty assert themselves and their autonomy: they point out their deviant behaviour in an academic space, and in doing so, they *resist*.

1.4. The fourth and final subtheme, *my work as a source of meaning*, connotes the value that academic work brings to their lives. Experiences of meaning make managing a little easier, a little lighter, bringing buoyancy to their efforts of staying afloat. Value comes in many forms and is experienced both momentarily and more constantly. Momentary gratification is derived from academic work and expressed in positive emotions including happiness, feeling stimulated, passionate, and congruence. Meeting students and teaching is one way in which faculty derive direct value from their work. One interviewee expresses the joy they feel when students demonstrate their understanding:

I think it’s really fun to teach because I see how students, they come in and don’t know anything and then. I see that they’ve understood something [-] for me it’s a thrill. (5023)

Besides the satisfaction and enjoyment faculty experience in relation to their students, value is also derived from faculty experiences of feeling stimulated:

[-] I would probably say that I, that I enjoy it very much eh I think it’s very inspiring what I am doing it’s very varied there are lots of different elements. [-] (5010)

Experiences of gratifying work also seem connected to the experience of engaging in questions and pursuing issues, which are important to the individual. Academic work is a means to act upon and live out their own values, and in doing so, experience congruence between what they believe and what they do:

the aspect that is regarding research is also very fulfilling, [-] that interaction and doing work, engaging in a social question that drives you and being able to be in a classroom, to interact with people, to learn from one another and teach and listen [-] That is, I would say, that amazing driving the lure of academia. (5025)

Value is also derived in a diffuse way, particularly in expressions of social responsibility and collective concern. Here, value is framed in terms of what one contributes or aspires to contribute, revealing a prosocial inclination. Prosocial tendencies are exemplified through feelings of social responsibility, the desire to help others, expressions of solidarity, beliefs that one has an impact, and the desire to have an impact. Faculty express a sense of responsibility for others, particularly their students and colleagues:

[-] I work a lot with health and it’s also something that you hope has some effect that you contribute something so that things get better for people, of course I don’t think my research directly saves lives or anything, all the doctoral students and students I supervise, a lot of people are better off because I do what I do. (5013)

Feelings of responsibility for others contribute to a sense of belonging, being important, and ultimately to being part of something greater than just themselves. Clearly, faculty see themselves as members of society, thus assuming social responsibility in various ways. A prosocial orientation is also reflected in beliefs that what they do is a contribution in some way, for example towards future knowledge-production. Here, an individual describes how their work enables other researchers’ work:

And then to see the other researchers when they come here that they’re like happy and think “we can do so much thanks to what you’ve come up with”. It’s also a kind of reward that it’s appreciated ((-)) that they in turn can do research which leads to advances. (5019)

Not only do faculty consider themselves as contributors to something other than themselves, but there is also an expressed desire to have an impact, which can be realized through their academic roles. For instance, an interviewee explains how they work to facilitate opportunities for other faculty at their department to use the research time allocated in their contracts, but in reality, never have the time to take advantage of. They also express the importance they placed on participating in these interviews:

[-] it’s nice in a way to hear that many of us have prioritized this because hopefully it can show what the situation looks like for many of us. (5017)

## I’m not yet where I’m supposed to be

This theme articulates the experiences of faculty as they orient themselves in time, from this phase of liminality (being between insecurity and security), involving aspiration, ambition, reflection, evaluation, and adjustment. This theme highlights the tensions and negotiations that faculty are working through, through both orientation (working towards an imagined future) and (dis)orientation (trying to make sense of the past and present). The theme embodies a strong future orientation among faculty who focus on striving towards an imagined future; one characterized as more secure than their current situations. Simultaneously, this process also involves appraisals of their pasts and presents, and faculty reflections and self-evaluations, as they make sense of their accumulated experiences at this point in time, with all the adjustments made along the way. This theme includes three subthemes: 2.1) *constructing and constructions of the future*, 2.2) *Should I have known better?*, and 2.3) *downward and upward adjustment*.

2.1. The subtheme *constructing and constructions of the future* accounts for how faculty share a strong sense of directedness: they express being goal-driven, with desires, hopes, aspirations, and ambitions for their futures. Not all desires are expressed as realistic, but hopes for secure employment, funding security, and financial security are examples of how faculty imagine their futures. Moreover, faculty are striving to close the gap between now and the future, which is constructed in terms of increased security. Striving to close this gap is exemplified by the various milestones ahead and the efforts they make to realize them. Many are concrete, including promotions, for instance, but also getting a permanent employment contract, or being able to use one’s research time for *research*.

Constructions of the future centre around a shared notion: that the future holds a turning point. Faculty express a clear idea as to what that point *should* look like: it should be a secure “place” where investments made in their academic careers are not perceived as wasted. This is a place to build on and make use of their knowledge, skills, and expertise. Ultimately, it is about maintaining or retaining their academic selves, but without the current insecurity. This place can be in or outside academia. The specific outcome of this turning point is portrayed as more or less realistic (or likely), depending on the discipline and subject area. Some express scepticism about finding a job outside academia allowing them to retain their academic selves:

[-] I find it very difficult to see my professional experience and knowledge I find it difficult to see where I could benefit from them. I mean because what I do has, of course, there’s a social relevance to it for example in my research. But, the way I do it, I find it hard to imagine that anyone would take a chance that “yes! we need him for this”. So I think there’s this inability both for me and many other academics to see that you can be useful or fit in with other jobs so to speak. Even though at the same time I think I probably would. I mean I could have another job, where my knowledge and skills would fit in or whatever. (5016)

Others have developed themselves in such a way to make their anticipated turning point or transition a smooth one, with more possibilities and choices:

I’ve learned as much as possible. Programming is pretty easy and since I thought it’s good for academia in the future because there’s so much with data, but also in industry it’s really important to be able to code. So I’ve learned as much as possible about it and so-called big data and IT and ICT as well so it could be good to find some job in data or programming. (5023)

2.2. Articulating their imagined futures, these constructions are influenced by how faculty reflect upon and understand their current and past selves and experiences. A tension exists in reconciling their choices with the current insecurity, evident in this subtheme *Should I have known better?*. While faculty do acknowledge their successes, they grapple with trying to make sense of why they still experience insecurity, through self-evaluation and social comparison. Individual responsibility permeates the material as faculty accept responsibility for their past choices and decisions, yet the extent of which varies, from self-blame to having made a “fully-informed” decision.

One of the ways in which faculty make sense of their current situations is by attributing their liminality to faulty decision-making in the past. Hindsight leads faculty to take individual responsibility, blaming themselves for not being strategic enough or making poor choices:

[-] I’ve come to the conclusion that maybe I made a mistake, I made a mistake early on [-] I should’ve studied at some other university, a better one, [-] better resources, higher prestige, I should’ve invested more from the beginning, I spent too much time hanging out with friends and having fun, took a long time to write my thesis [-] I was late to the game which means that it what I experience now, now I’m still a productive researcher and kind of well-qualified but I’m kind of behind [-] (5003)

Social comparison plays into their more self-critical stance, revealing an underlying belief that had they made “better” decisions, they would not be experiencing this insecurity; instead, they would be in a *secure* situation. Social comparison also adds to the tension of reconciling their choices, especially when certain decisions may be regarded as having made sacrifices:

[-] I didn’t apply for much money here, I went a different way, I followed the money then, since I did my PhD, I’m not part of the so-to-say inbred Swedish academy, where everyone knows everyone and people stay at the same workplace all their lives, I’ve been mobile, for better or worse, a lot has been bad with this too, but a lot has been good. [-] (5009)

While some blame themselves for their choices, others are not so heavy-handed. Instead, faculty may assert that they do not regret their choices, but it is clear that some decisions were made without a full understanding or awareness of the cognitive and affective implications of their choice:

[-] If someone had said that “when you’re over 40 you still won’t have had a permanent position”. Then I wouldn’t have done it. (5016)

Even in cases where decisions are framed as fully-informed, faculty recognize the consequences these decisions have had for their careers.

I mostly don’t care about that, I do what I want ((laughs)). But then I have to accept that it’s not going to, you won’t progress as quickly. Then you can be criticized for it, but you know that it’s, the rules are so that or yeah, not the rules but the informal ones, because the only thing that counts is results, so if you work twice as much, you’ve got a greater chance of getting twice as many publications and then a greater chance of becoming professor faster, that’s how it is. There’s nothing strange about that. But then you have to choose whether you want to run that race or some other race, I’ve chosen another race (5021).

2.3. *Downward and upward adjustment*, the third subtheme, accounts for the ways in which faculty have adjusted and adapted themselves over time (sometimes out of necessity, sometimes more naturally), involving a practice of acknowledgement, reconciliation and acceptance (to varying degrees). Faculty experience challenges to their beliefs framed in terms of notions of themselves—their self-concepts—but also in terms of their beliefs about *right* and *wrong*. While many of these experiences of adjustment imply a downward adjustment, where faculty must accept that “things are not as they should be”, some experiences are of upward adjustment as well. Beliefs and values that may have been unwavering in the past, change or are redefined, illustrating how over time, one’s life experiences and circumstances can encourage growth and changing perspectives.

Adjustment is required where there is a disconnect between expectations and reality. Faculty convey that loyalty or citizenship behaviours are not rewarded in academia: these are not formal grounds for promotion, nor contribute to their professional qualifications. Nonetheless, faculty still perceive situations where their contributions are not recognized nor rewarded, which are conveyed as violations of reciprocity. Thus, adjustment involves reconciling one’s cognitive beliefs with one’s affective beliefs. Others also articulate a frustration with academia in that as a system of meritocracy, it only rewards certain contributions.

How do I describe my work situation? If you focus on just one day, things are really good [-] but if you look at the bigger picture, perhaps more in terms of my career, then I’m not as satisfied. When you have a tenure-track position, it’s temporary. So I can find it a bit annoying that you step up and support the department, ((-)) and yet you’re still not employed permanently (5020).

In other words, faculty must adjust their beliefs about norms of reciprocity to fit the academic context, and forgo certain beliefs about fairness.

A disconnect between expectations and reality is also evident as faculty grapple with experiences of “wasting” their competence. Faculty share strong beliefs about how best to utilize one’s research education and experience, and an administrative burden does not align with these beliefs. This downward adjustment, where their beliefs about what it means to do academic work are challenged, triggers frustration and creates dissonance in that one must act in a way that feels inherently wrong for the individual, but also for society, as society bears the costs.

When it comes to the self-concepts of faculty, downward adjustment communicates a kind of settling, the need to adapt to and accept something *less than*. Their self-concepts include qualities such as being dedicated, engaged, thorough, and competent. However, faculty have experiences of making downward adjustments, thereby challenging their sense of selves. These experiences of adjustment require adaptation, yet they also entail an internal struggle, particularly of acceptance. For instance, they repress aspects of themselves which they view positively, to avoid conflicts with colleagues:

[-] you have to try to find your own way of dealing with it [-] some conflicts arise in the workplace and some people who are halfway to burnout aren’t always easy to deal with [-] I don’t even try to get involved anymore because it’s not possible anymore, there ended up being too many conflicts. [-] (5005)

Other faculty voice similar experiences, where they experience an incongruence between their self-concepts and actions/behaviours which their positions of liminality seem to require:

[-] I’m a researcher but I don’t do research. (5009).

Experiences of liminality challenge and erode faculty notions about academia, and these experiences contribute to reshaping faculty members’ values. In particular, academia has lost its prior appeal when faculty recognize having reached a limit, one where what was once acceptable is experienced as no longer acceptable. Simply put, tolerance has been eroded over time, and old notions about academia have shifted into new ones. These are characterized by the realization that they deserve better working and employment conditions.

Conceptualizations of success are another adjustment that faculty explicitly and implicitly articulate. Success is portrayed as something dichotomous; faculty are either successful or unsuccessful, despite the advancements they have made in their careers. What defines success is linked to what is needed or required to stay in academia. Over time, definitions of success have become more limited, narrower, consisting of a select few measurable indicators of performance. For instance, faculty may perceive themselves as having checked the necessary boxes that characterize “being successful”, yet there is still an uncertainty as to whether it is enough, meaning that their conceptualization of success must be adjusted again.

Experiences of insecurity can be catalysts for new perspectives and outlooks, and such adjustments can be referred to as upward adjustments. It may seem provoking to refer to them as such, and this is not to detract from the experiences of struggle and frustration, but rather to bring attention to the temporal component, specifically how the compounding of experiences over time can incite shifts in beliefs, even values. Upward adjustment can also account for a reorganization of priorities, where life circumstances have contributed to adjustments, and over time, shifts in their values, regarding their professional ambitions. Being (and becoming) a parent, for instance, is one of the major life changes referred to as bringing about adjustments on which faculty reflect positively.

## Discussion

This qualitative study sought to investigate experiences and expressions of insecurity and PWB among faculty with experiences of insecurity. Two themes (*Staying afloat?* and *I’m not yet where I’m supposed to be*) were developed and illustrate how PWB among faculty is a dynamic, ongoing process of negotiation and balancing, involving individual responsibility, accountability, and adjustment.

*Staying afloat?* illustrates the *ebb and flow* of PWB, specifically regarding environmental mastery and autonomy. The theme reveals certain inherent contradictions that faculty experience; they have chosen academia based on a perceived conduciveness to pursuing their interests and values, yet in striving for continuity in academia, this complicates their experiences of competence as faculty try to manage. Similarly, the extent to which faculty are independent seems contingent upon this chosen environment, and upon their approaches to managing in this context. Part of managing involves decision-making and weighing the possible outcomes, in particular, whether these will affect their continuity (either by extending or curtailing their time in academia). Ultimately, faculty must balance their desire for a future in academia, with acting independently. Taken together, faculty seem motivated to pursue certain endeavours, even when the pursuit of these may inherently limit opportunities elsewhere.

Patterns of managing are in line with previous research where faculty treat time as a commodity (Ylijoki & Mäntylä, 2003), but also where time reallocation involves personal sacrifices (Nicholls et al., 2022). These have consequences for the social fulfilment of faculty, limiting faculty capacity to cultivate and maintain positive social relations. Other patterns involve restrained resistance, offering some relief for faculty trying to manage (Archer, 2008), yet there are risks involved. The consequences of such acts remain unknown as they may lead to divergent outcomes: some may bring continuity, while others may inevitably lead to outcomes which are counterproductive to continuity.

Taken together, *Staying afloat?* embodies important psychosocial factors of academia and academic work. In fact, these may be comparable to facets of the effort-reward imbalance model, where work-related effort is balanced out with work-related rewards (Siegrist, 1996). Not only do the subthemes reveal various demanding aspects of work within academia which faculty face, but also the ways in which they experience rewards, echoing previous research where the authors characterize work in academia as “bittersweet” (Knights & Clarke, 2014). An imbalance between effort and reward appears present, with implications for faculty health (Kinman, 2019; Tanimoto et al., 2023). This is made evident as some faculty teeter on the verge of work-related ill-health. Faculty must carefully manage their work and related responsibilities, while keeping health-related consequences at bay, but the tenuousness of the situation means there is no guarantee that faculty will be successful. In fact, *“It’s about trying not to get totally burnt out”* suggests that burnout and work engagement may occur in parallel, among faculty who are highly motivated and engaged in their work, yet without perhaps knowing their own limits. Indeed, it was not unusual for faculty to share experiences of adverse work-related health during the interviews, specifically an awareness of health-related changes or developments in relation to work.

Theme 2, *I’m not yet where I’m supposed to be*, reflects the individual’s ongoing process of fulfilment, where PWB dimensions including purpose in life, personal growth and self-acceptance are in focus. Overall, the theme reveals that faculty express a strong sense of purpose in life and personal growth, two dimensions of PWB which most closely reflect eudaimonia (Ryff & Singer, 2008). Faculty demonstrate intentionality in their efforts to progress in their careers, and to shape their futures in ways allowing them to retain their academic selves, to some degree. This process in itself leads to a sense of purposefulness, where faculty derive meaning from the experience of striving to align themselves with their goals and values (Rosso et al., 2010). Importantly, it is the process, the passing of time coupled with accumulating life experiences which brings about personal growth among faculty with experiences of insecurity. While it is clear that downward adjustments may trigger dissonance, or negative emotions including frustration, they elicit self-reflection and sense-making as faculty integrate these experiences into their sense of self. Over time, this can lead to learning, development, and an increased awareness and understanding of oneself, all qualities characterizing personal growth (Ryff, 1989).

Experiencing career-related challenges or setbacks and adjusting to these has implications for faculty attitudes towards themselves and their past selves. Subthemes *Should I have known better?* and *downward and upward adjustment* illustrate the complexities of self-acceptance, a dimension of PWB related to self-efficacy (Ryff, 1989). Faculty perceive themselves as agentic actors assuming individual responsibility for their situations, making it challenging at times to maintain positive self-regard. In reflecting critically on how one’s career has developed, faculty seem to perpetuate the notion that they as individuals have the capacity to shape their academic careers, so long as they are strategic enough. Given the liminal position they occupy, and that they have yet to reach that anticipated turning point, this period seems characterized by constrained self-acceptance: faculty are more attentive to their failings than their strengths. This evokes what is popularly known as the *arrival fallacy* (Ben-Shahar, 2009) as faculty look to the future, which is constructed as no longer insecure, as a positive turning point, bringing about increased self-regard and harmonizing self-acceptance.

### Integrating insecurity and well-being

Together, the themes reveal that the insecurities of academia have implications for faculty experiences of PWB. Some of the complexities embedded in the coexistence of insecurity and well-being relationship may be obscured by quantitative approaches. The themes empirically show that the various experiences faculty have reflect how certain PWB dimensions are more salient during this time. Clearly, transitional periods may bring certain PWB dimensions to the fore.

Faculty pursue academia with the belief that they can continue to conduct meaningful work there. Their experiences of deriving meaning and purpose from their work lends greater legitimacy to this belief, thereby solidifying the notion that staying in academia is conducive to a strong sense of purpose in life. This occurs despite the struggles faculty experience, and the subsequent consequences they incur regarding their health and personal lives, substantiating prior research (Soren & Ryff, 2023). Simultaneously, their future orientation, marked by a desired sense of security, reflects what may be considered to be the *ultimate reward*. While for many this involves getting a permanent contract, others articulate a *sense* of security, which they have yet to reach, but is framed as the reward for their current, ongoing efforts.

These findings demonstrate how PWB is dynamic, constantly shifting and changing with the passing of time, reflecting a view and approach to eudaimonia which Shir and Ryff (2022) advocate for. Particularly, transitional periods elicit a reorganization of an individual’s constellation of PWB dimensions, impacting the prevalence and salience of certain dimensions, reflecting other empirical studies of PWB (e.g., Trainor et al., 2020). Thus, while PWB is quantifiable, this fine-grained investigation of individual experiences of PWB, within a highly educated group, qualitatively adds an understanding of how individuals in insecure positions negotiate constraints and conduits of PWB.

### Methodological considerations and future research

This study was conducted within the context of Swedish academia, and while perceptions of insecurity in academia may differ across countries and welfare systems (Klug et al., 2024), the implications of such insecurity for faculty PWB may translate to academia as a whole. Not only are faculty likely motivated by similar values and rewards, but overall, the shared principles and values which govern academia as a meritocracy generally translate across borders.

An important methodological consideration regards the power dynamics in the interviews, considering the hierarchical structure of academia. Differences in academic seniority between the interviewers and interviewees may involve faculty being uncomfortable with sharing certain experiences. However, the power dynamics could shift during the interview, as the interviewer had scientific knowledge about the topic in question, while the interviewee possessed personal opinions and experiences. Such shifts likely contributed to a balanced atmosphere during the interviews. Also, the interviewers were able to establish a friendly yet professional rapport during the interviews, and faculty were generally forthcoming when sharing.

Our strategic approach to selecting the participant group required setting strict exclusion criteria. Consequently, individuals with low self-ratings of job insecurity, and/or who no longer worked in academia were excluded. Hence, our findings do not capture the PWB of individuals no longer working in academia, nor the PWB of those individuals who have attained a sense of security.^7^ Regarding the former, future research should investigate how individuals negotiate changes in PWB post-academia, if and how they retain their academic selves, and whether some harmonization of PWB *actually* occurs. Regarding the latter, it is plausible that individuals currently in academia without persisting perceptions of insecurity, recognize themselves in our results, having reached that highly-anticipated sense of security. Here too, future studies should investigate if and how PWB is recalibrated after faculty have reached this crucial point in their careers, and where their sights turn to next, if new goals are set. Future qualitative studies might consider focusing on faculty narratives of insecurity during one’s academic career, and quantitative studies might benefit from longitudinal designs to map PWB as experiences of insecurity in academia change over time.

## Conclusion

This study examined faculty experiences of insecurity and eudaimonic well-being in Swedish academia through RTA, contributing to a contextualized understanding of PWB. In addition to showing that academia, with its constraints and opportunities, impacts faculty experiences of PWB, time seems to play into their PWB experiences. Importantly, the study illustrates how PWB among faculty with experiences of insecurity is a dynamic process, with the potential to both constrain and facilitate opportunities for fulfilment. Also, the findings reveal how faculty appraise academic work as somewhat disconnected from the employment and working conditions in academia, despite these being what make doing the work possible. Moreover, while insecurity in academia may involve various adverse consequences for faculty, PWB in eudaimonic terms appears to be more balanced than previous, quantitative research seems to suggest. Understanding how faculty navigate the insecurities of their positions and academia to stay afloat and strive towards a desired, secure position, requires a focus beyond mental health problems. It requires considering individuals’ negotiations of the constraints and conduits of their positive psychological functioning.

## Notes

1. Approximately half of the respondents *within* each of the five profiles expressed a willingness to participate in the follow-up interview study.

2. Job insecurity scores that did not qualify as moderate to high ranged from 1 to 2.9 on a Likert response scale.

3. The use of the term *participant group* instead of *sample* is recommended to align with practices within the qualitative paradigm (Braun & Clarke, 2022).

4. Median was 100%, mean was 93%.

5. Note that the term *position* refers to a place, not to an employment position.

6. These non-work time examples reflect traditional working hours, which may not entirely coincide with *when* academic work is conducted.

7. Here, attaining a sense of security refers to individuals who had previously rated their job insecurity as high, but at the start of this interview study, reported that they no longer experienced high job insecurity.

## References

[cit0001] Archer, L. (2008). The new neoliberal subjects? Young/er academics’ constructions of professional identity. *Journal of Education Policy*, 23(3), 265–13. 10.1080/02680930701754047

[cit0002] Bautista, T. G., Roman, G., Khan, M., Lee, M., Sahbaz, S., Duthely, L. M., Knippenberg, A., MacIas-Burgos, M. A., Davidson, A., Scaramutti, C., Gabrilove, J., Pusek, S., Mehta, D., & Bredella, M. A. (2023). What is well-being? A scoping review of the conceptual and operational definitions of occupational well-being. *Journal of Clinical and Translational Science*, 7(1), e227. 10.1017/cts.2023.648 38028344 PMC10643923

[cit0003] Bazzoli, A., & Probst, T. M. (2022). Taking stock and moving forward: A textual statistics approach to synthesizing four decades of job insecurity research. *Organizational Psychology Review*, 12(4), 507–544. 10.1177/20413866221112386

[cit0004] Ben-Shahar, T. (2009). *The pursuit of perfect: How to stop chasing perfection and start living a richer, happier life*. McGraw-Hill.

[cit0005] Braun, V., & Clarke, V. (2006). Using thematic analysis in psychology. *Qualitative Research in Psychology*, 3(2), 77–101. 10.1191/1478088706qp063oa

[cit0006] Braun, V., & Clarke, V. (2022). *Thematic analysis: A practical guide*. Sage Publications.

[cit0007] Castellacci, F., & Viñas-Bardolet, C. (2021). Permanent contracts and job satisfaction in academia: Evidence from European countries. *Studies in Higher Education*, 46(9), 1866–1880. 10.1080/03075079.2019.1711041

[cit0008] Finlay, L. (2021). Thematic analysis: The ‘Good’, the ‘Bad’ and the ‘ugly’. *European Journal for Qualitative Research in Psychotherapy*, 11, 103–116.

[cit0009] Fischmann, G., De Witte, H., Sulea, C., Vander Elst, T., De Cuyper, N., & Iliescu, D. (2022). Validation of a short and generic qualitative job insecurity scale (QUAL-JIS). *European Journal of Psychological Assessment*, 38(5), 397–411. 10.1027/1015-5759/a000674

[cit0010] Hellgren, J., Sverke, M., & Isaksson, K. (1999). A two-dimensional approach to job insecurity: Consequences for employee attitudes and well-being. *European Journal of Work & Organizational Psychology*, 8(2), 179–195. 10.1080/135943299398311

[cit0011] Keyes, C. L. M., Shmotkin, D., & Ryff, C. D. (2002). Optimizing well-being: The empirical encounter of two traditions. *Journal of Personality & Social Psychology*, 82(6), 1007–1022. 10.1037/0022-3514.82.6.1007 12051575

[cit0012] Kinman, G. (2019). Effort-reward imbalance in academic employees: Examining different reward systems. *International Journal of Stress Management*, 26(2), 184–192. 10.1037/STR0000128

[cit0013] Klug, K., Selenko, E., Hootegem, A., Sverke, M., & De Witte, H. (2024). A lead article to go deeper and broader in job insecurity research: Understanding an individual perception in its social and political context. *Applied Psychology*, 73(4), 1–34. 10.1111/apps.12535

[cit0014] Knights, D., & Clarke, C. A. (2014). It’s a bittersweet symphony, this life: Fragile academic selves and insecure identities at work. *Organization Studies*, 35(3), 335–357. 10.1177/0170840613508396

[cit0015] Låstad, L., Berntson, E., Näswall, K., Lindfors, P., & Sverke, M. (2015). Measuring quantitative and qualitative aspects of the job insecurity climate: Scale validation. *Career Development International*, 20(3), 202–217. 10.1108/CDI-03-2014-0047

[cit0016] Låstad, L., Tanimoto, A. S., & Lindfors, P. (2021). How do job insecurity profiles correspond to employee experiences of work-home interference, self-rated health, and psychological well-being? *Journal of Occupational Health*, 63(1), e12253. 10.1002/1348-9585.12253 34322976 PMC8319028

[cit0017] Levitt, H. M., Motulsky, S. L., Wertz, F. J., Morrow, S. L., & Ponterotto, J. G. (2017). Recommendations for designing and reviewing qualitative research in psychology: Promoting methodological integrity. *Qualitative Psychology*, 4(1), 2–22. 10.1037/qup0000082

[cit0018] Miron, A. M., Branscombe, N. R., Ball, T. C., McFadden, S. H., Haslam, C., & Truxillo, D. M. (2022). An interpretative phenomenological analysis of social identity transition in academic retirement. *Work, Aging and Retirement*, 8(1), 82–97. 10.1093/workar/waab018

[cit0019] Nicholls, H., Nicholls, M., Tekin, S., Lamb, D., Billings, J., & Serraino, G. F. (2022). The impact of working in academia on researchers’ mental health and well-being: A systematic review and qualitative meta-synthesis. *PLOS ONE*, 17(5), e0268890. 10.1371/JOURNAL.PONE.0268890 35613147 PMC9132292

[cit0020] Pautz, M. C., & Vogel, M. D. (2020). Investigating faculty motivation and its connection to faculty work-life balance: Engaging public service motivation to explore faculty motivation. *Journal of Public Affairs Education*, 26(4), 437–457. 10.1080/15236803.2020.1776076

[cit0021] Rosso, B. D., Dekas, K. H., & Wrzesniewski, A. (2010). On the meaning of work: A theoretical integration and review. *Research in Organizational Behavior*, 30, 91–127. 10.1016/j.riob.2010.09.001

[cit0022] Ryan, R. M., & Deci, E. L. (2001). On happiness and human potentials: A review of research on hedonic and eudaimonic well-being. *Annual Review of Psychology*, 52(1), 141–166. 10.1146/annurev.psych.52.1.141 11148302

[cit0023] Ryff, C. (1989). Happiness is everything, or is it? Explorations on the meaning of psychological well-being. *Journal of Personality & Social Psychology*, 57(6), 1069–1081. 10.1037/0022-3514.57.6.1069

[cit0024] Ryff, C., & Singer, B. (2008). Know thyself and become what you are: A eudaimonic approach to psychological well-being. *Journal of Happiness Studies*, 9(1), 13–39. 10.1007/s10902-006-9019-0

[cit0025] Segerbäck, J. (2023). *To do research or not: Job insecurity among employees with a doctoral degree in academia*. Stockholm University.

[cit0026] Shir, N., & Ryff, C. D. (2022). Entrepreneurship, self-organization, and eudaimonic well-being: A dynamic approach. *Entrepreneurship Theory and Practice*, 46(6), 1658–1684. 10.1177/10422587211013798

[cit0027] Siegrist, J. (1996). Adverse health effects of high-effort/low-reward conditions. *Journal of Occupational Health Psychology*, 1(1), 27–41. 10.1037/1076-8998.1.1.27 9547031

[cit0028] Soren, A., & Ryff, C. D. (2023). Meaningful work, well-being, and health: Enacting a eudaimonic vision. *International Journal of Environmental Research and Public Health*, 20(16), 6570. 10.3390/ijerph20166570 37623156 PMC10454804

[cit0029] Swedish Association of University Teachers and Researchers & National Junior Faculty. (2021). *Uncertainty- a reality for junior researchers in Sweden* (Swedish Association of University Teachers and Researchers and the National Junior Faculty) https:// sulf.se/app/uploads/2021/03/sulf-njf-uncertainty-xxx-a-reality-forjunior-researchers-in-sweden-finalreport.pdf.

[cit0030] Tanimoto, A. S., Richter, A., Bujacz, A., & Lindfors, P. (2025). Are profiles of job insecurity associated with health-related indicators among faculty in Swedish academia? *Scandinavian Journal of Psychology*, 66(1), 85–97. 10.1111/sjop.13064 39187960 PMC11735251

[cit0031] Tanimoto, A. S., Richter, A., & Lindfors, P. (2023). How do effort, reward, and their combined effects predict burnout, self-rated health, and work-family conflict among permanent and fixed-term faculty? *Annals of Work Exposures and Health*, 67(4), 462–472. 10.1093/annweh/wxac094 36617194 PMC10119699

[cit0032] Trainor, L. R., Crocker, P. R. E., Bundon, A., & Ferguson, L. (2020). The rebalancing act: Injured varsity women athletes’ experiences of global and sport psychological well-being. *Psychology of Sport & Exercise*, 49, 101713. 10.1016/j.psychsport.2020.101713

[cit0033] Urbanaviciute, I., Roll, L. C., Tomas, J., & De Witte, H. (2021). Proactive strategies for countering the detrimental outcomes of qualitative job insecurity in academia. *Stress & Health*, 37(3), 557–571. 10.1002/smi.3023 33377270

[cit0034] Ylijoki, O.-H., & Mäntylä, H. (2003). Conflicting time perspectives in academic work. *Time & Society*, 12(1), 55–78. 10.1177/0961463X03012001364

